# Mechanism and inhibition of human UDP-GlcNAc 2-epimerase, the key enzyme in sialic acid biosynthesis

**DOI:** 10.1038/srep23274

**Published:** 2016-03-16

**Authors:** Sheng-Chia Chen, Chi-Hung Huang, Shu-Jung Lai, Chia Shin Yang, Tzu-Hung Hsiao, Ching-Heng Lin, Pin-Kuei Fu, Tzu-Ping Ko, Yeh Chen

**Affiliations:** 1 Department of Biotechnology, Hungkuang University, Taichung, Taiwan; 2 Taiwan Advance Biopharm (TABP), Inc., Xizhi City, New Taipei City, Taiwan; 3 Institute of Biological Chemistry, Academia Sinica, Taipei, Taiwan; 4 Department of Medical Research, Taichung Veterans General Hospital, Taichung, Taiwan; 5 Division of Critical Care & Respiratory Therapy, Department of Internal Medicine, Taichung Veterans General Hospital, Taichung, Taiwan

## Abstract

The bifunctional enzyme UDP-GlcNAc 2-epimerase/ManNAc kinase (GNE) plays a key role in sialic acid production. It is different from the non-hydrolyzing enzymes for bacterial cell wall biosynthesis, and it is feed-back inhibited by the downstream product CMP-Neu5Ac. Here the complex crystal structure of the N-terminal epimerase part of human GNE shows a tetramer in which UDP binds to the active site and CMP-Neu5Ac binds to the dimer-dimer interface. The enzyme is locked in a tightly closed conformation. By comparing the UDP-binding modes of the non-hydrolyzing and hydrolyzing UDP-GlcNAc epimerases, we propose a possible explanation for the mechanistic difference. While the epimerization reactions of both enzymes are similar, Arg113 and Ser302 of GNE are likely involved in product hydrolysis. On the other hand, the CMP-Neu5Ac binding mode clearly elucidates why mutations in Arg263 and Arg266 can cause sialuria. Moreover, full-length modelling suggests a channel for ManNAc trafficking within the bifunctional enzyme.

The sialic acids are a family of nine-carbon sugar derived from neuraminic acid^1^ (Fig. 1a). These negatively charged sugars are frequently found on mammalian cell surface as the terminal component of glycoconjugates and play very important functions in cellular recognition^2^. Excessive production of sialic acid-rich glycoproteins is often linked to metastatic cancer^3^,^4^. The sialic acids are also involved in influenza virus binding (hemagglutinin) and release (neuraminidase)^5^. Although limited to those associated with higher animals, many bacteria produce and incorporate sialic acids into their surface glycoconjugates to evade host immune response^6^.

Sialic acid biosynthesis in mammals starts by converting UDP-GluNAc into UDP and ManNAc, followed by phosphorylation of ManNAc at the sixth position (Fig. 1b). Catalysis of both reactions is carried out by the bifunctional enzyme GNE, which in human is composed of ~720 amino acid residues and divided into the epimerase part (~400 aa) and the kinase part (~300 aa)^7^. The equivalent enzymes in bacteria lack the kinase moiety but show about 35% sequence identity in the epimerase part. In a downstream reaction ManNAc (or ManNAc-6P) combines with phosphoenolpyruvate to produce Neu5Ac (or Neu5Ac-9P), which is eventually activated by CTP for incorporation into surface glycoconjugates^7^,^8^ (Fig. 1b).

Being a key enzyme that catalyzes the rate-limiting step of sialic acid biosynthesis, GNE plays an important role in regulation of cell-surface sialyation level by binding to the downstream product CMP-Neu5Ac. The feedback inhibition is highly positively cooperative and it does not affect the ManNAc kinase activity^9^. Defective GNE inhibition by CMP-Neu5Ac causes cytoplasmic accumulation and increased excretion of free sialic acid. Sialuria is an autosomal dominant disorder which is related to GNE mutation in one of the two arginine residues 263 and 266 (R263L, R266Q or R266W)^10^. On the other hand, by mediating cell-cell recognition, sialic acids are important in the development of nervous system^11^. Reduced sialylation levels can result in neuromuscular disorders, including the recessively inherited disease hereditary inclusion body myopathy (HIBM)^12^. Mutations that caused HIBM have been shown to spread over both epimerase and kinase moieties of GNE^13^,^14^,^15^.

The kinase part of GNE forms a homodimer^16^,^17^, whereas the full-length enzyme can form a dimer or tetramer, depending on the presence of UDP-GlcNAc and CMP-Neu5Ac^18^. Despite the limited sequence identity of less than 20%, the hydrolyzing and non-hydrolyzing UDP-GlcNAc epimerases are believed to share a common protein fold and similar catalytic mechanisms^19^. Functionally, the non-hydrolyzing enzyme participates in teichoic acid biosynthesis, and it is positively regulated by UDP-GlcNAc^20^. Crystal structures of the non-hydrolyzing enzyme showed that each monomer comprises two Rossmann (N and C) domains^20^,^21^,^22^. The binding of UDP-GlcNAc to an allosteric site triggered domain closure and activates the enzyme by forming a proper substrate-binding pocket.

GNE has been expressed in mammalian and insect cells, as well as in slime mold^23^. However, considering the possible flexibility of the bifunctional enzyme, we focused on the epimerase part instead of full-length GNE. To understand the mechanism by which CMP-Neu5Ac inhibits GNE, and to find out the difference between non-hydrolyzing and hydrolyzing UDP-GlcNAc epimerase, we crystallized the epimerase part of human GNE and determined its structure in the presence of substrate and inhibitor. The precise interactions of the enzyme with UDP and CMP-Neu5Ac helped explaining the mechanism of catalysis and inhibition. The proposed mechanism was supported by activity measurements of selected active-site mutants.

## Results

### GNE forms a closed tetramer in the presence of UDP-GlcNAc and CMP-Neu5Ac

**Figure 2:**
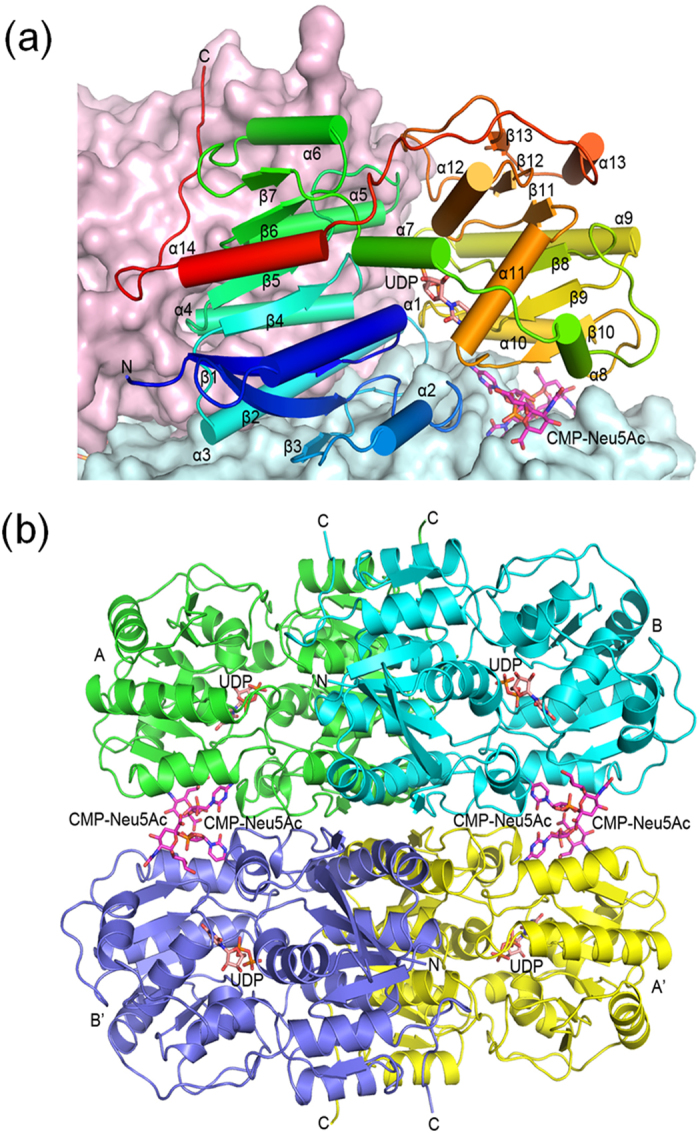
Structure of the epimerase part of GNE. (**a**) A monomer is shown as a ribbons diagram, with cylinders and arrows representing α-helices and β-strands. The model is colored from N-terminus to C-terminus by a spectrum from blue to red. Also shown are surface representations of the other monomers in the same dimer (colored light pink) and the tetramer-forming dimer (light blue). The bound UDP in the active site is depicted as a pink stick model. Two CMP-Neu5Ac molecules at the dimer-dimer interface are colored magenta. (**b**) The tetramer is shown as a ribbons diagram where the subunits are in four different colors. Monomers A and B form a dimer and monomers A’ and B’ form another. The stick models of UDP and CMP-Neu5Ac are colored pink and magenta.

The crystal structure of the epimerase part of human GNE was determined at 2.7-Å resolution by single-wavelength anomalous diffraction (SAD) method using Se-Met enriched protein. Refinement statistics are summarized in Table 1. The protein folds into 13 β-strands and 14 α-helices (Fig. 2a). Each asymmetric unit comprises four monomers (denoted A, B, C and D) in two homodimers. The dimeric structure is similar to that of non-hydrolyzing enzyme. When the dimer is compared with the ligand-bound *M. jannaschii* epimerase, the RMSD is 2.3 Å between 618 Cα atoms, indicating a closed conformation (Supplementary Fig. S1a). The dimer is formed by the N domain, principally involving non-polar side chains on helices α3, α4 and α5 (Fig. 2a). The buried surface area by dimer formation is 1860 Å^2^ on each monomer.

In addition, each dimer further forms a tetramer with a crystallographic dyad-related dimer (Fig. 2b), burying 1280 Å^2^ surface area on each monomer and showing the 222 point-group symmetry as observed in hemoglobin. Direct protein-protein interactions at the tetramer interface mostly involve the N domain. In the center of tetramer a hydrophobic core is formed adjacent to helix α3, by the side chains of Val76, Leu90 and Val93. Surrounding this patch are several salt bridges like Arg77-Glu79’ and Lys92-Asp97’. Further away from the tetramer center, two pairs of CMP-Neu5Ac molecules intercalate between the dimers (Fig. 2b), burying an additional 390 Å^2^ surface area on each monomer. Interactions with CMP-Neu5Ac involve the C domain.

**Figure 3:**
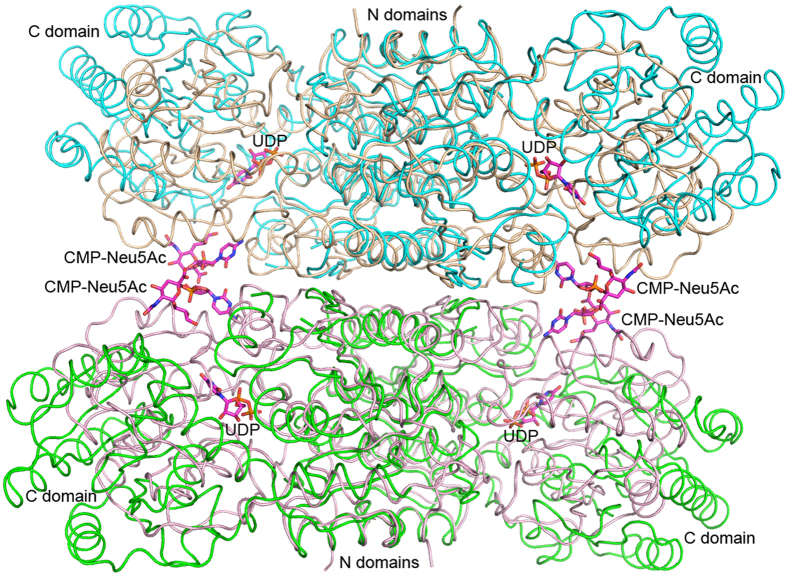
Open and closed conformations of the GNE tetramer. The tetramer is shown as a worm tracing diagram, with the two dimers colored light pink and salmon. Two copies of the dimer of nonhydrolyzing epimerase from M. jannaschii (PDB 4NEQ) are superimposed on GNE by using the N domain as in supplementary Fig. S1b. These are colored cyan and green.

Unlike the non-hydrolyzing epimerase, the closed conformation of human GNE is probably not active. The substrate-binding site is excluded from the bulk solvent by the association of surrounding loops, especially loops β3-α3 and β9-α10, which are longer than the equivalents in the non-hydrolyzing enzymes. Because GNE uses the N domain to form a tetramer, movement of the C domain to make a more open conformation is necessary to allow product release and new substrate binding. If the human GNE dimer is superimposed on the free *M. jannaschii* dimer by using the N domain, the RMSD will be 1.6 Å between 344 Cα atoms (Supplementary Fig. S1b). If the GNE monomer assumes the open conformation as does the free *M. jannaschii* enzyme, the C domain will move away from each other (Fig. 3).

### The binding mode of UDP reveals unique interactions with the hydrolyzing epimerase

**Figure 4:**
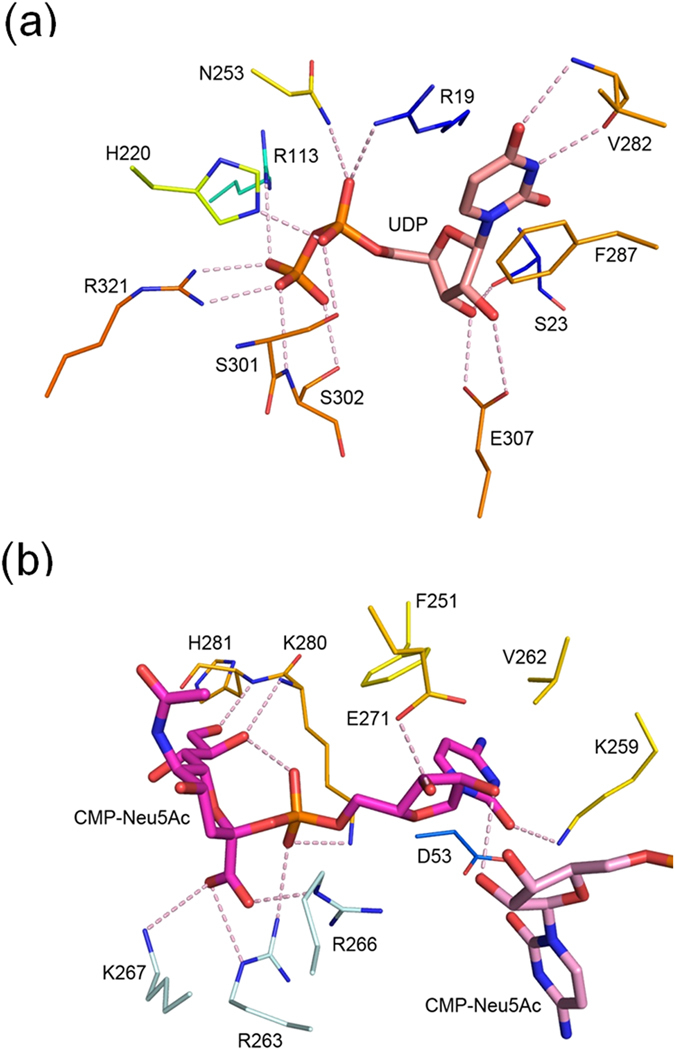
UDP and CMP-Neu5Ac interactions with GNE. (**a**) The bound UDP is shown as a thick stick model with carbon atoms colored in pink. The participating amino-acid residues are depicted as thin sticks in which the carbon atoms are color coded as in Fig. 2b. Potential hydrogen bonds are shown as pink dashes. (**b**) The two bound CMP-Neu5Ac molecules are colored magenta and light pink. The amino-acid residues are colored as in (**a**) except for three from another monomer, which are colored light blue.

Only UDP was observed in the active site of each monomer despite the use of UDP-GlcNAc in crystallization. Presumably the substrate had been converted to ManNAc and UDP before it was trapped in the crystal. In fact, a ManNAc molecule was bound to a surface pocket formed by helices α1, α2 and α11 of monomer C (Supplementary Fig. S3). The binding mode of UDP is similar to that of non-hydrolyzing enzyme (Fig. 4a), with virtually identical interactions for the uracil and ribose moieties. The base is sandwiched between the side chains of Arg19 and Phe287, while making two hydrogen bonds to the backbone of Val282. The two hydroxyl groups of ribose are hydrogen bonded to the side chains of Ser23 and Glu307. The pyrophosphate forms salt bridges to Arg19, Arg113 and Arg321. It also interacts with His220 and Asn253 through hydrogen bonds, as well as the two consecutive Ser301 and Ser302 at the N-terminus of helix α12.

Structural comparisons of the non-hydrolyzing and hydrolyzing epimerases indicate that the latter enzyme has more extensive direct interactions with UDP. For example, in the *M. jannaschii* enzyme, the ribose forms double hydrogen bonds with Glu294 (Glu307 in GNE) but there is no equivalent bond to that of Ser23 in GNE (Supplementary Fig. S4). Despite the similar disposition in both enzymes, the β-phosphate interacts with Arg208 adjacent to His207 (His220 in GNE) at the C-terminus of strand β8, instead of Arg321 from the β12-β13 loop in GNE. The β-phosphate also forms a hydrogen bond to the backbone N of Gly290 (Cys303 in GNE) instead of the Ser302 side chain. More importantly, the interactions of pyrophosphate with Arg113 and Asn253 in GNE are replaced by hydrogen bonds to the O4 and O6 atoms of “allosteric” UDP-GlcNAc in the non-hydrolyzing enzyme (Supplementary Fig. S5). The stronger pyrophosphate interactions may be related to the hydrolyzing characteristics of the GNE-catalyzed reaction, without requirement of a second UDP-GlcNAc for activity.

### The role of catalytic base is served by conserved acidic residues

**Figure 5:**
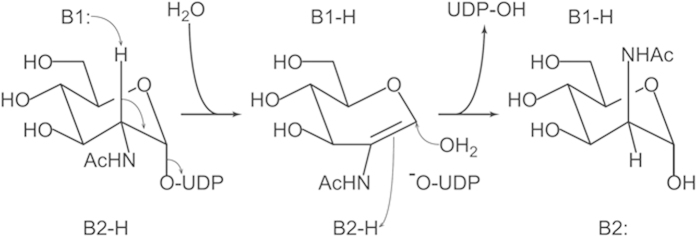
Catalytic mechanism of hydrolyzing UDP-GlcNAc 2-epimerase. B1 and B2 are two catalytic bases. B1 deprotonates the C2 atom on one side, forming a 2-acetamidoglucal intermediate. B2 re-protonates the same atom from opposite side when an activated water molecule attacks the C1 atom.

It has been proposed that both non-hydrolyzing and hydrolyzing UDP-GlcNAc epimerase catalyze the reaction by using 2-acetamidoglucal as an intermediate^24^. The catalysis starts by deprotonation at the C2 atom of GlcNAc, followed by elimination of UDP and formation of the C = C double bond. In the non-hydrolyzing enzyme, the intermediate is re-protonated on the other side and re-united with UDP. In the hydrolyzing enzyme the reaction proceeds by syn-hydration of 2-acetamidoglucal. The catalysis requires two different bases B1 and B2, which are located on the opposite sides of the sugar ring (Fig. 5). Three acidic residues, equivalent to Asp112, Glu134 and Asp143 in GNE, are supposed to function as the base. However, the crystal structure of an enzyme-product complex from *Escherichia coli* (PDB 1VGV; Supplementary Fig. S5a) shows that they are all “above” the sugar ring, probably serving as B1 but not B2. The most likely candidate is Asp112, which is strictly conserved among 500 homologous sequences of hydrolyzing UDP-GlcNAc epimerase found by NCBI BLAST. The equivalent Asp95 of the *E. coli* enzyme is hydrogen bonded to the 2-NH and 3-OH groups of ManNAc.

Subsequent experiments showed that the mutant D112A retained about 2% specific activity of the wildtype enzyme (Table 2). Its k_cat_ value was reduced by more than 150 fold. The other two mutants E134A and D143A were completely inactivated. Although not strictly conserved, Glu134 may be important in binding to the 4-OH group of ManNAc (Supplementary Fig. S5b). An equivalent glutamine (43 cases in 500 homologues) or histidine (17 cases) residue, but not alanine, can play the same role. Asp143 is also strictly conserved among hydrolyzing UDP-GlcNAc epimerase, but its equivalent in the *E. coli* enzyme is Glu131, which does not interact directly with the sugar. However, an alternative model of the substrate UDP-GlcNAc suggests a possible hydrogen bond between the side chain of Asp143 and the 2-NH group of the sugar (Supplementary Fig. S6a). The acetyl group may stack with the Arg321 side chain and form a hydrogen bond to Gln322. In this arrangement, Asp143 will be 3.6 Å from the C2 atom of GlcNAc and able to serve as the first catalytic base. Furthermore, because of its disposition, Asp143 can have access to the other side of the sugar ring via mediating solvent molecules and participate in a proton relay system to serve indirectly as a general acid/base in catalysis (Supplementary Fig. S6b).

### A third base can be involved in the hydrolyzing epimerase reaction

In addition to Asp112, there are eight other strictly conserved residues in both hydrolyzing and non-hydrolyzing UDP-GlcNAc 2-epimerases (Supplementary Fig. S7). The conformational flexibility and lack of side chain in Gly111, Gly136 and Gly182 account for their structural roles. The hydrogen bond between the His132 imidazole and the Gly111 carbonyl groups should be important in maintaining the special conformation of Gly111 and the precise orientation of Asp112. The side chain of Arg147 forms a salt bridge to that of Asp112, and thus ensures the ionization state of the catalytic base. At the N-terminus of helix α1, the Arg19 side chain not only stacks with the uracil base of UDP but also makes a hydrogen bond to the α-phosphate. Only two residues His220 and Ser301 are strictly conserved in the C domain. Both are involved in binding to the α-phosphate.

Interestingly, when the sequences of hydrolyzing and non-hydrolyzing enzymes are compared, the β-phosphate-binding residues Arg113 and Ser302 of GNE are found conserved in the hydrolyzing enzymes. The corresponding residues, Thr96 and Gly289 of the *M. jannaschii* enzyme, are also conserved in the non-hydrolyzing enzymes. The GNE mutant R113A was completely inactivated, while S302A retained about 13% activity (Table 2). As shown above, Arg113 of the hydrolyzing enzymes takes over the role of allosteric UDP-GlcNAc in the non-hydrolyzing enzymes. By forming a direct salt bridge to the β-phosphate, Arg113 also makes UDP a better leaving group. The binding of β-phosphate to the Ser302 side chain instead of backbone NH group further provides a possibility for the β-phosphate and Ser302, by serving as a catalytic base, to activate a water molecule for attacking the C1 atom of the intermediate. Specifically, a proton can be removed from the water molecule via the OH group of Ser302 by the β-phosphate (Supplementary Fig. S6b). This hypothesis is consistent with the reduction of k_cat_ and K_M_ values by 15 fold and 3 fold in the mutant S302A (Table 2).

### The cooperative binding mode of CMP-Neu5Ac explains the cause of sialuria

The allosteric inhibitor CMP-Neu5Ac is bound in an L-shaped conformation, forming an internal hydrogen bond between the CMP phosphate and the 8-OH of Neu5Ac (Fig. 4b). The cytosine base is sandwiched between the side chains of Asp53 and Val262. It also makes a T-stacking interaction with Phe251 and a hydrogen bond to Lys259. The 2-OH of the ribose interacts with its equivalent of the other bound CMP-Neu5Ac molecule, and the 3-OH is hydrogen bonded to Glu271. The phosphate group makes two additional hydrogen bonds to the side chains of Lys280 and Arg263* (residues from the other monomer in the A–B’ or A’–B pair are denoted by asterisks). The carboxyl group of Neu5Ac, a characteristics of this negatively charged nine-carbon sugar, interacts with the three positively charged side chains of Arg263*, Arg266* and Lys267*. The terminal 8-OH and 9-OH groups of Neu5Ac, not found in most other sugars, are hydrogen bonded to the backbone N of Lys280 and His281. All interactions with the inhibitor are contributed by residues from the C domain except Asp53.

As mentioned above, the C domain must move away from one another to make an open active site for catalytic turnover. However, the CMP-Neu5Ac binding site will be disrupted by the C-domain movements. Therefore, at high CMP-Neu5Ac concentrations, the GNE tetramer assumes the closed, inactive, conformation to maintain the favored interactions with the inhibitor. In addition, because CMP-Neu5Ac binds in pairs at each site, the inhibition is positively cooperative with a Hill coefficient of 4.1^9^. It is likely related to the stringent feedback regulation of GNE activity, and clearly explains why single mutations at Arg263 or Arg266 can cause sialuria. The presence of a ManNAc molecule at the interface of N and C domains may further impede conformational changes, but the possibility of such direct product inhibition remains to be investigated. By comparison, the regulation mechanism of the non-hydrolyzing epimerase is very different, in which allosteric binding of UDP-GlcNAc triggers domain closure into an active conformation.

### Full-length modeling of GNE suggests a possible epimerase-kinase channel

In the crystal, the C-terminal loop is docked adjacent to the dimer interface but makes few specific interaction. Its average temperature factor of 118 Å^2^ is significantly higher than the overall 78 Å^2^. The two Phe389 near the end are separated by 17 Å, whereas the two Gln380 at the beginning are 49 Å apart. Because the two Gly405 in the ManNAc kinase dimer are separated by 73 Å (according to PDB 2YHY), it is likely that the flexible C-terminal loop of the epimerase part swings outward to connect with the kinase part in a full-length GNE dimer. A model was thus constructed by aligning the dyad axes of the epimerase and kinase dimers (Supplementary Fig. S2). The epimerase active site of one monomer may be closer to the kinase active site of the other monomer. The 25-residue loop may extend from helix α14 across the epimerase-kinase interface, which may be hydrophilic with only a few specific bonds like an inter-monomer salt bridge of Glu326-Lys532, and it may also shield a possible channel from bulk solvent for ManNAc trafficking (Supplementary Fig. S2).

On the other hand, based on the full-length GNE model, the precise locations of 87 HIBM-causing mutants in the epimerase part were analyzed (Supplementary Table S1). The results are generally consistent with previous data^25^. Most mutations occurred on the protein surface, except a few are now involved in possible interactions between the epimerase part and the kinase part (Supplementary Fig. S8). For example, in the full-length dimer, Arg335 of one monomer may be close to Asp715 of the other, and its positively charged side chain can also associate with the C-terminal dipole of the last α-helix. The uncharged bulky indole group in the mutant R335W may abort all these interactions. Although previous studies suggest that the kinase and epimerase parts function independently^7^,^8^, because substrate channeling to the kinase part may be affected by mutants in the epimerase part, the kinase activity can also be reduced. The quantitative correlation between the two functional parts of GNE, however, remains to be further investigated.

## Discussion

In this study we determined the crystal structure of the epimerase part of human GNE and investigated its catalytic mechanism. The epimerase showed a higher k_cat_ value than did the full-length enzyme (12 vs 0.33 s^−1^; Table 2) whereas the K_M_ values were similar (33 vs 26 μM)^26^. The faster turnover rate is likely due to the absence of the kinase part. In the full-length enzyme, although the epimerase can undergo open/close conformational changes, the kinase moiety may nonetheless pose some restraints on its flexibility. Besides, the presence of a connecting segment (380–405) and a possible ManNAc trafficking channel in the full-length enzyme can slow down the rate of substrate/product exchange. For a comparison, the bacterial hydrolyzing UDP-GlcNAc epimerase SiaA from *Neisseria meningitidis* showed a k_cat_ value of 4.7 s^−1^, which is also larger than that of full-length human GNE^27^.

Judging by their arrangement in the active site and drastically reduced activity of the mutants, Asp112 and Asp143 of human GNE appear to function as the catalytic bases in the epimerase reaction. In non-hydrolyzing enzymes these two acidic residues have their equivalents. In the two-base mechanism of catalysis, after the first base B1 obtains a proton from C2 of the sugar, some solvent molecule may mediate the action of second catalytic base B2 to protonate the 2-acetamidoglucal on the other side of the sugar ring (Fig. 5). The precise pathway of proton transfer from one side to the other awaits further investigation. More importantly, in hydrolyzing UDP-GlcNAc epimerase a third base B3 may come to play in activating a water molecule to attack the C1 atom (Supplementary Fig. S6b). A similar mechanism has been proposed for O-GlcNAc transferase, in which the serine side chain is deprotonated by the pyrophosphate to attack the sugar^28^. In contrast, since equivalent amino acids to Arg113 and Ser302 are not found in the non-hydrolyzing enzymes, UDP reunites with the sugar after epimerization.

The crystal structure of GNE also contains CMP-Neu5Ac, a downstream product in sialic acid biosynthesis that binds to the allosteric regulatory site. The inhibitor binding mode, in which Arg263 and Arg266 are involved, not only explains why mutations at these two sites can cause sialuria but also provides an opportunity to design novel nucleoside-based drugs to regulate sialic acid production. Because extensive sialylation is associated with tumor cells and metastasis, GNE inhibitors can be used as anticancer drugs. Successful reduction of cell surface sialylation level has been achieved by inhibition of ManNAc kinase^29^. Regarding the epimerase part, based on the CMP-Neu5Ac binding mode, it might be possible to design a new drug by crosslinking the ribose moieties of the two nucleosides to mimic a pair of bound cytidine at the dimer-dimer interface. The dinucleotide analogue can be more potent than the natural GNE inhibitor by simultaneously occupying two CMP-Neu5Ac binding sites.

A previous study by Ghaderi *et al.*^18^ showed that GNE is in an equilibrium state between monomer, dimer, tetramer and higher oligomers, whereas the presence of UDP-GlcNAc and CMP-Neu5Ac favors tetramer formation^16^. Judging by the significant level of intracellular UDP-GlcNAc and its stronger effect on GNE tetramer formation, the authors postulated that the enzyme may function as a tetramer. Consistently, our crystal structure shows that the tetramer is stabilized by a large interface area of 1280 Å^2^ per monomer, with an additional 390 Å^2^ involved in CMP-Neu5Ac binding. Structural comparison with non-hydrolyzing epimerase suggests that the tetrameric assembly of GNE does not prevent domain opening to make the active site solvent-accessible, although the inhibitor-bound enzyme is locked in a tightly closed conformation (Fig. 3). However, the possibility of GNE functioning as a dimer cannot be excluded because gel-filtration experiments indicated a dimeric form of the apo-enzyme (data not shown), which can be more flexible for efficient substrate/product exchange during catalysis.

With no more than 20% sequence identity, the hydrolyzing and non-hydrolyzing UDP-GlcNAc 2-epimerase share a common dimeric architecture and similar catalytic mechanism, but the hydrolyzing enzymes are for sialic acid biosynthesis, different from those for making bacterial cell wall. First, they do not need an allosteric UDP-GlcNAc molecule for activity. Equivalent bonds to the pyrophosphate group are provided by the protein. Second, they make the β-phosphate a better leaving group by salt bridging to Arg113. At the same time, the phosphate group can serve as a third base to activate a water molecule for hydrolysis, thanks to the presence of the mediating Ser302. In addition, human GNE can form a tetramer and is subject to regulation by CMP-Neu5Ac, but the bacterial enzymes (both hydrolyzing and non-hydrolyzing) lack the corresponding subunit interface and allosteric site.

## Methods

### Cloning, expression and purification

The gene encoding the UDP-GlcNAc 2-epimerase domain was PCR amplified using human cDNA as the template and was then cloned into the pET21a(+) vector, to be expressed as a His_6_-tagged recombinant protein in *Escherichia coli* BL21 (DE3) cells. The transformed BL21 (DE3) cells were grown to an OD_600_ of 0.4–0.6 in LB medium with 100 mg L^−1^ ampicillin and induced with 50 μM IPTG for 14–16 h at 16 °C. Expression of Se-Met protein was carried out by using M9 medium supplemented with 40 mg L^−1^ seleno-l-methionine. The cells were harvested by centrifugation, and the cell pellet was suspended in a binding buffer of 50 mM Tris-HCl pH 8.0, 500 mM NaCl and 5 mM imidazole. After sonication, the lysates were centrifuged at 30,000 × g for 1 h. The clarified supernatant was applied to Ni-NTA resin pre-equilibrated with the binding buffer. Impurities were removed with a wash buffer of 50 mM Tris-HCl, pH 8.0, 500 mM NaCl and 10 mM imidazole, and the bound 2-epimerase was eluted with a 0–200 mM linear gradient of imidazole. Fractions containing the 2-epimerase were pooled, concentrated and loaded onto a Superdex-200 size-exclusion column (GE Healthcare) equilibrated with a gel filtration buffer of 50 mM Tris-HCl, pH 8.0, 100 mM NaCl, 5% glycerol and 2 mM TCEP (Supplementary Fig. S9). The fractions containing the His_6_-tagged 2-epimerase domain of GNE were pooled and concentrated to 5 mg ml^−1^ for crystallization screening.

### Crystallization and data collection

For complex formation, the recombinant human UDP-GlcNAc 2-epimerase was incubated in 5 mM UDP-GlcNAc and 5 mM CMP-Neu5Ac for 16-18 h at 4 °C. Crystals of the ternary complex were grown by sitting-drop vapor-diffusion from 0.2 M ammonium acetate, 0.1 M HEPES, pH 7.1, 19% PEG 3350, transferred to mother liquor supplemented with 25% glycerol as cryoprotectant, and flash frozen in liquid nitrogen. The crystals belong to the space group *C*2, have typical unit-cell dimensions of a = 106 Å, b = 98 Å, c = 154 Å, β = 96° and contain four protein molecules in the asymmetric unit. X-ray diffraction data were collected at the National Synchrotron Radiation Research Center (NSRRC) BL13B1 in Taiwan. The data were indexed and integrated using the HKL2000 processing software^30^.

### Structure determination and refinement

Phase angles for the Se-Met complex crystals were obtained by using Se-SAD data and Phenix^31^. The initial protein model was improved with Phenix Autobuild^31^ followed by cycles of manual building with Coot^32^. Computational refinement was performed with Phenix^31^ and Refmac^33^. Stereochemical libraries were prepared using phenix.elbow. The model geometry was monitored on the MolProbity server^34^. The data collection and structure refinement statistics are given in Table 1. Although the Ramachandran plot shows a few outliers, most are near the allowed regions (Supplementary Fig. S10). SA omit maps clearly indicate the presence of UDP and CMP-Neu5Ac in the crystal (Supplementary Fig. S11). Coordinates have been deposited in the Protein Data Bank with the accession code 4ZHT. All figures of the protein structure and electron-density map were generated by using PyMOL (www.pymol.org)

### Site-specific mutagenesis

The mutants were generated by using the QuikChange kit (Stratagene), with the parental expression plasmid pET21a-GNE as a template. All resulting mutants were verified through DNA sequencing. The mutants were expressed and purified as described for the wild-type enzyme, yielding a single band on SDS-polyacrylamide gel electrophoresis.

### Activity measurements

The UDP-GlcNAc 2-epimerase assays of the recombinant GNE and its mutants in this study follow the procedures described before with partial modification^9^. Briefly, the activity assays were performed in 50 mM Tris buffer system (pH 7.5) containing 10 mM MgCl_2_, 0.2 mM of NADH, 2 mM of phosphoenol pyruvate (PEP), 2 unit of pyruvate kinase, 2 unit of lactic dehydrogenate, 10 nM to 300 nM of UDP-GlcNAc. The protein amount for each reaction of the wildtype and mutant GNE depended on the production rate fitting into the initial rate. The reaction was initiated by addition of WT-GNE, D112A, R113A, E134A, D143A, or S302A with 0.5 μM, 10 μM, 15 μM, 15 μM, 5 μM, and 5 μM, respectively. The final volume of each reaction was 100 μL. The initial velocities were determined via measuring the decrease in extinction at 340 nm at 37°C per minute during the reaction time. The kinetic parameters were measured by Eadie-Hofstee plots with equation V = −K_M_ (V/[S]) + V_max_. The k_cat_ value was calculated by V_max_/[Enzyme] using enzyme concentration described above. The mutants R113A, E134A, and D143A did not show detectable activity. Specific activity in this study was defined as the nano-molarity of product obtained per second per micro-molarity of enzyme with 50 nM of UDP-GlcNAc as the substrate. Each data point represents the average from triplicate experiments.

## Additional Information

**How to cite this article**: Chen, S.-C. *et al.* Mechanism and inhibition of human UDP-GlcNAc 2-epimerase, the key enzyme in sialic acid biosynthesis. *Sci. Rep.* **6**, 23274; doi: 10.1038/srep23274 (2016).

## Supplementary Material

Supplementary Information

## Figures and Tables

**Figure 1:**
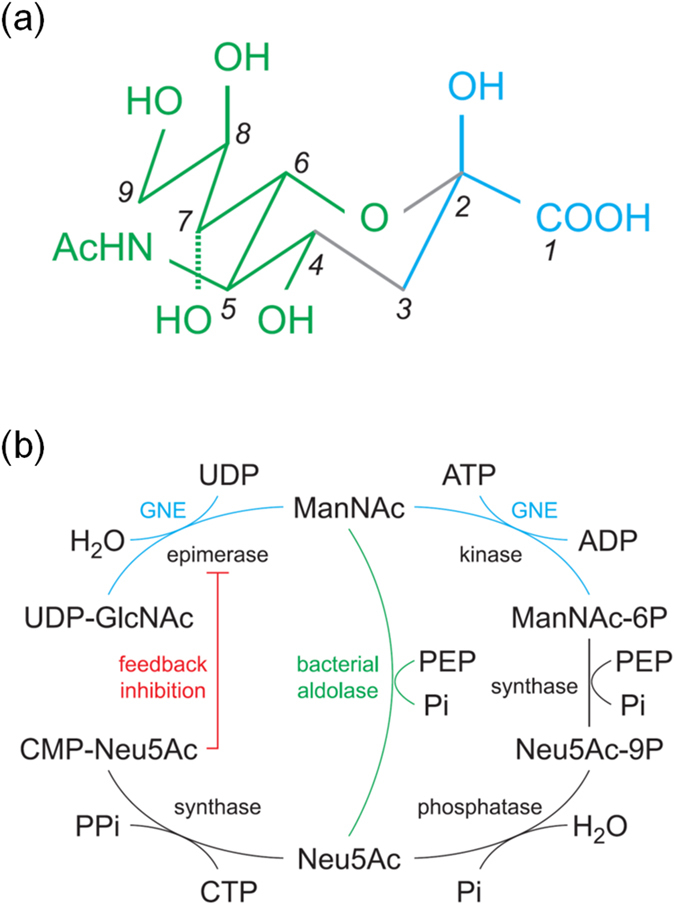
Structure and biosynthesis of sialic acid. (**a**) The structure of β-Neu5Ac, a commonly occurring sialic acid, is shown as a schematic diagram. In an α-anomer the configuration at C2 is inverted, with the OH and COOH switched. For another type of sialic acid, 2-keto-3-deoxynoic acid (Kdn) the N-acetyl group at C5 is replaced by a hydroxyl (OH) group. The green part on the left comes from ManNAc and the cyan part on the right is from PEP. (**b**) The biosynthesis of sialic acid starts by converting UDP-GlcNAc into UDP and ManNAc by an epimerase. In mammals ManNAc is phosphorylated at C6 by a kinase before further reaction with PEP. In bacteria the phosphorylation step is bypassed. The bifunctional GNE (cyan) catalyzes both epimerase and kinase reactions. Neu5Ac is eventually activated by CTP, and the product CMP-Neu5Ac is a potent inhibitor for the epimerase part of GNE.

**Table 1:** X-Ray Data Collection, Phasing and Refinement Statistics.

Data collection	
Wavelength (Å)	0.9791
Space group	C2
Unit cell a, b, c (Å)	106.04, 98.10, 154.72
	β = 96.04°
Resolution (Å)	30.0–2.70 (2.80–2.70)
Total observations	326520 (31587)
Unique reflections	43536 (4327)
Redundancy	7.5 (7.3)
R_merge_ (%)*	9.7 (59.1)
I/σ (I)	14.8 (2.9)
Phasing	
No. of Se sites	13
Figure of merit	0.43
No. of autobuilt residues	1231 (76%)
Refinement	
Resolution (Å)	30.0–2.70 (2.76–2.70)
No. of reflections (Working/Free)	41334/3091
R_work_/R_free_ (%)†	18.9/22.5 (29.3/33.0)
R.m.s.d. bond lengths (Å)	0.010
R.m.s.d. bond angles (°)	1.6
Ramachandran plot (%)‡	
Favored	95.5
Allowed	3.4
Outlier	1.1
Average B-values (Å^2^)/ No. of atoms	
Protein	77.7/12071
UDP	57.1/100
CMP-Neu5Ac	59.3/164
ManNAc	94.7/15
Water	67.3/331

Values in parentheses are for the highest resolution shells.

^*^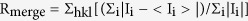 for all equivalent reflections.

^†^R_work_ and R_free_ were calculated as 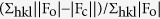 for 95% data used in the refinement and 5% data that were excluded.

^‡^Checked by using MolProbity^34^.

**Table 2:** Kinetic Parameters and Activity of GNE Mutants.

	k_cat_ (s^−1^)	K_M_ (μM)	k_cat_/K_M_ (s^−1 ^μM^−1^)	Specific activity (μM s^−1 ^μg^−1^)	relative activity (%)*
WT	11.8 ± 2.0	33.1 ± 4.2	0.355	1.01 ± 0.08	100
D112A	0.076 ± 0.003	0.5 ± 0.2	0.152	0.023 ± 0.002	2.3
R113A	N.D.†	N.D.^†^		N.D.^†^	
E134A	N.D.†	N.D.†		N.D.†	
D143A	N.D.†	N.D.†		N.D.†	
S302A	0.791 ± 0.033	12.1 ± 0.9	0.065	0.130 ± 0.008	12.9

^*^Calculated by using specific activity.

^†^Not detected.
