# Supplementary material for: Mechanism and inhibition of human UDP-GlcNAc 2-epimerase, the key enzyme in sialic acid biosynthesis
Source: Sci Rep. 2016 Mar 16;6:23274. doi: 10.1038/srep23274 (PMC4793188; doi:10.1038/srep23274)
Supplement: Supplementary Information [file srep23274-s1.pdf]

Supporting Information

Mechanism and inhibition of human UDP-GlcNAc 2-epimerase (GNE), the key enzyme in sialic acid biosynthesis

Sheng-Chia Chen [a,b¥], Chi-Hung Huang [a,b¥], Shu-Jung Lai [c], Chia Shin Yang [a,b], Tzu-Hung Hsiao [d], Ching-Heng Lin [d], Pin-Kuei Fu [e], Tzu-Ping Ko [c,*], and Yeh Chen [a,*]

[a] Department of Biotechnology, Hungkuang University, Taichung, Taiwan
[b] Taiwan Advance Biopharm (TABP), Inc., Xizhi City, New Taipei City, Taiwan
[c] Institute of Biological Chemistry, Academia Sinica, Taipei, Taiwan
[d] Department of Medical Research, Taichung Veterans General Hospital, Taichung ,Taiwan
[e] Division of Critical Care & Respiratory Therapy, Department of Internal Medicine, Taichung Veterans General Hospital, Taichung, Taiwan

Supplementary Table S1. Mutation sites on the epimerase part of human GNE that cause inherited disorders. Mutants 58, 60 and 61 caused sialuria. All others caused HIBM.

No.	Amino acid substitution	Structural position	Localization of amino acids within GNE protein	Reference
1	E2G	Flexible N-terminus	surface	1
2	R8*	Flexible N-terminus	surface	1-3
3	R11W	β1 strand	surface	4
4	C13S	β1 strand	inside	2,5-7
5	A26P	α1 helix	inside	8
6	P27L	α1 helix	inside	3,9
7	P27S	α1 helix	inside	10
8	I28M	α1 helix	inside	11
9	M29T	α1 helix	inside	5,11
10	M29R	α1 helix	inside	11
11	E35K	$L_{\alpha1\text{-}\beta2}$	surface	2,12
12	P36L	$L_{\alpha1\text{-}\beta2}$	surface	13
13	E40K	β2 strand	surface	11
14	I51M	$L_{\eta1\text{-}\eta2}$	tetramer interface	2,12
15	M60V	α2 helix	inside	14
16	R71W	β3 strand	surface	1
17	G89R	α3 helix	dimer interface	11,15
18	G89S	α3 helix	dimer interface	3,11
19	R101C	α3 helix	tetramer interface	6
20	R101H	α3 helix	tetramer interface	11
21	I106T	β4 strand	inside	2
22	I128Ifs*6	$L_{\alpha4\text{-}\beta5}$	inside	3,11
23	R129Q	β5 strand	surface	3
24	R129*	β5 strand	surface	14
25	H132Q	β5 strand	inside	16,17
26	G135V	$L_{\beta5\text{-}\alpha5}$	inside	18
27	G136R	$L_{\beta5\text{-}\alpha5}$	inside	11
28	I142T	α5 helix	dimer interface	1
29	I150V	α5 helix	inside	19
30	Y156H	β6 strand	surface	14
31	H157fs	$L_{\alpha5\text{-}\beta6}$	inside	20

32	R162C	α6 helix	surface (kinase interface)	[9,21]
33	M171V	$L_{\alpha6-\beta7}$	dimer interface	[22]
34	D176V	η3 helix	surface	[6,7,12,16,17,23]
35	R177C	η3 helix	surface	[11,16]
36	I178N	β7 strand	inside	[11]
37	I178M	β7 strand	inside	[11]
38	L179F	β7 strand	inside	[24]
39	Y186C	α7 helix	inside	[19]
40	D187G	α7 helix	surface	[3,11]
41	N194Tfs*4	$L_{\alpha7-\alpha8}$	surface	[11]
42	I200F	α8 helix	inside	[13]
43	R202L	α8 helix	surface	[4]
44	W204*	α8 helix	inside	[1]
45	G206S	$L_{\alpha8-\beta8}$	surface	[10]
46	G206Vfs*3	$L_{\alpha8-\beta8}$	surface	[10]
47	D208N	$L_{\alpha8-\beta8}$	surface	[20]
48	D213V	$L_{\alpha8-\beta8}$	surface	[14]
49	V216A	β8 strand	inside	[4,25]
50	Q219K	$L_{\beta8-\eta4}$	inside	[11]
51	D225N	η4 helix	surface	[26]
52	F233S	α9 helix	inside	[3]
53	I241S	α9 helix	surface	[2,12,27,28]
54	R246W	β9 strand	surface	[1,11,12,18,27,29,30]
55	R246Q	β9 strand	surface	[1,10,26,28,31]
56	M261V	α10 helix	inside	[6]
57	M261I	α10 helix	inside	[20]
58	R263L	α10 helix	Allosteric site,	[32]
59	M265T	α10 helix	inside	[19]
60	R266Q	α10 helix	Allosteric site	[32]
61	R266W	α10 helix	Allosteric site	[32]
62	I270N	η5 helix	inside	[11]
63	I270T	η5 helix	inside	[11]
64	R277C	β10 strand	surface	[11,33]
65	R277G	β10 strand	surface	[11]
66	P283S	$L_{\beta10-\alpha11}$	surface	[7]
67	H293R	$L_{\alpha11-\beta11}$	surface	[34]
68	G295D	$L_{\alpha11-\beta11}$	inside	[3]

69	G295R	$L_{\alpha11\text{-}\beta11}$	inside	[11]
70	M297T	β11 strand	inside	[14]
71	I298T	β11 strand	inside	[1,2]
72	N300K	$L_{\beta11\text{-}\alpha12}$	inside	[35]
73	C303V	α12 helix	inside	[17]
74	C303*	α12 helix	inside	[26]
75	G304R	α12 helix	inside	[9]
76	R306Q	α12 helix	inside	[16]
77	A310P	η6 helix	inside	[27,30]
78	V315M	β12 strand	inside	[19]
79	N317D	β12 strand	inside	[19]
80	R321C	$L_{\beta12\text{-}\beta13}$	Active site	[3]
81	V331A	β13 strand	inside	[16]
82	H333R	β13 strand	surface	[8]
83	R335W	$L_{\beta13\text{-}\alpha13}$	surface (kinase interface)	[1,36]
84	L347P	$L_{\beta13\text{-}\alpha13}$	inside	[11]
86	Y361*	$L_{\alpha13\text{-}\alpha14}$	inside	[8]
86	Y361I	$L_{\alpha13\text{-}\alpha14}$	inside	[37]
87	I377Tfs*15	C-terminus	inside	[10]
88	D378Y	C-terminus	surface	[13,16,19]
89	L379H	C-terminus	surface	[38]
90	P390S	C-terminus	surface	[20]

Supplementary Figure S1. Dimer comparison with the hydrolyzing epimerase from *M. jannaschii*. (a) The closed dimer of the *M. jannaschii* enzyme (PDB 4NES; green) is superimposed on the GNE dimer (red). (b) The open dimer of PDB 4NEQ (cyan) is superimposed on GNE by using the N domain.

a

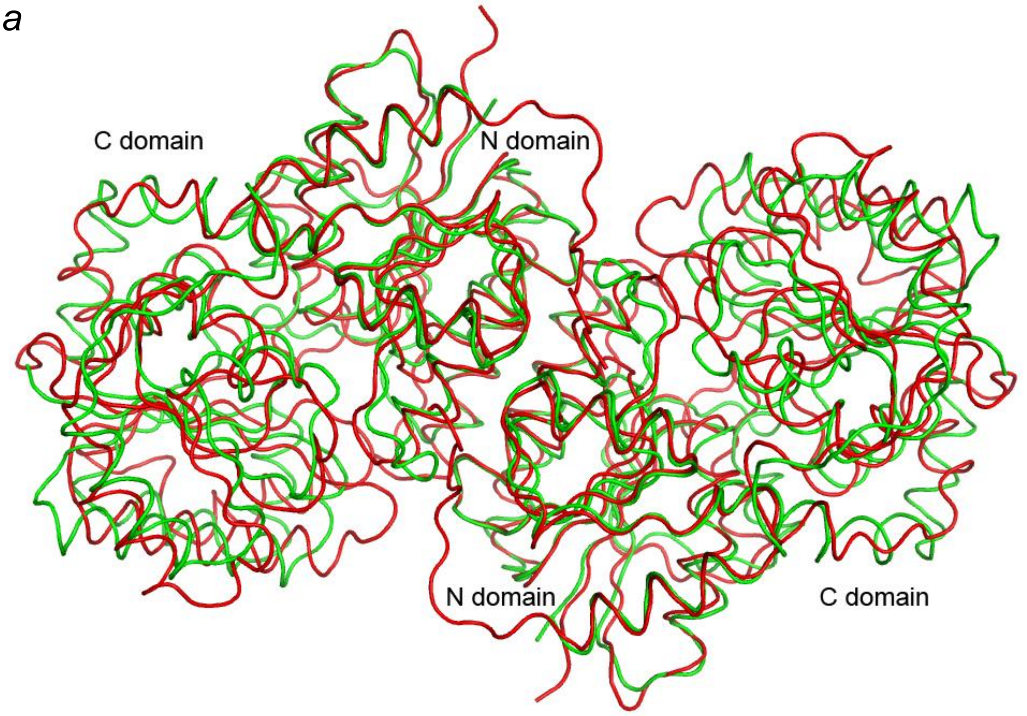

b

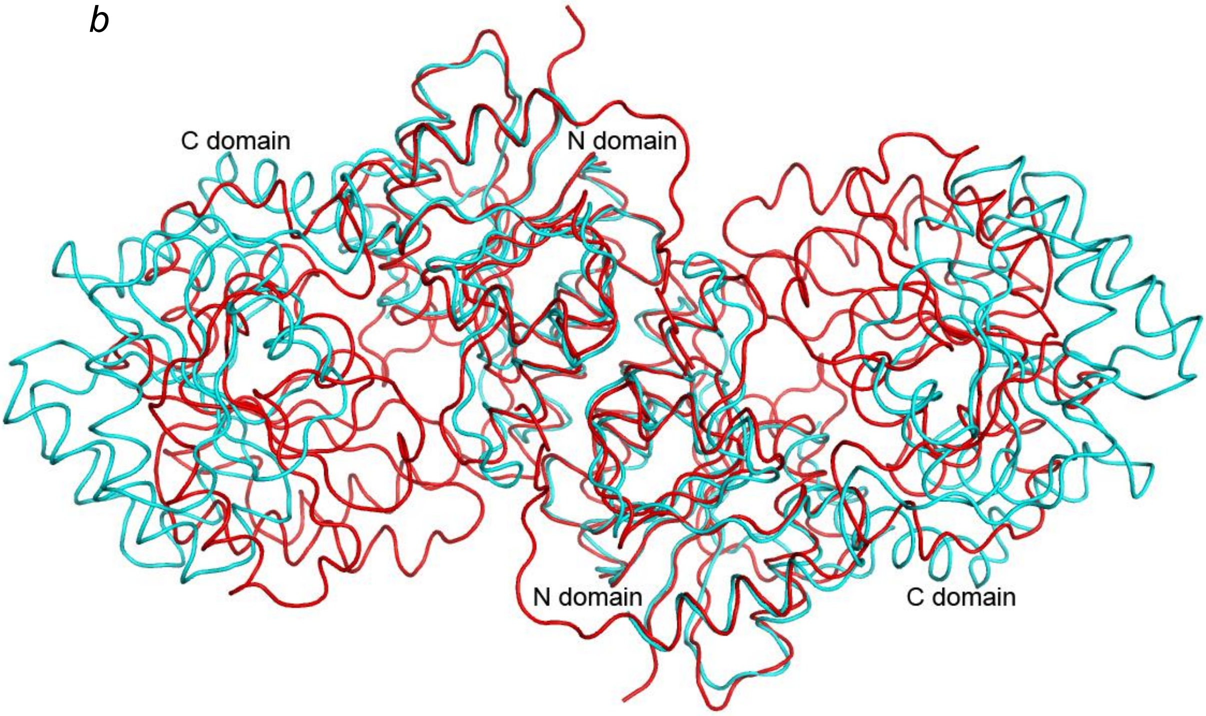

Supplementary Figure S2. Full-length model. The dimer of the kinase part of GNE (PDB 2YHY) was docked onto the tetramer of the epimerase part by dyad alignment and axial rotations. In (a) an epimerase monomer is color coded from N to C terminus and so is the corresponding kinase part. The linker of 380-405, colored magenta, is probably longer than shown here. Other monomers are colored pink. The bound ligands are shown as stick models. In (b) a surface presentation is shown with each monomer colored differently, except the linker of the green subunit is colored magenta. Shown in (c) are two different views of the full-length tetramer model, colored green and cyan. The same model is colored yellow in (d), where two open dimers of the *M. jannaschii* enzyme are superimposed on to the tetramer as in Figure S2 and colored green and cyan in place of those in (c). Semitransparent surface representations of the open dimers are also shown. A possible channel for ManNAc trafficking between epimerase and kinase is indicated by dashes.

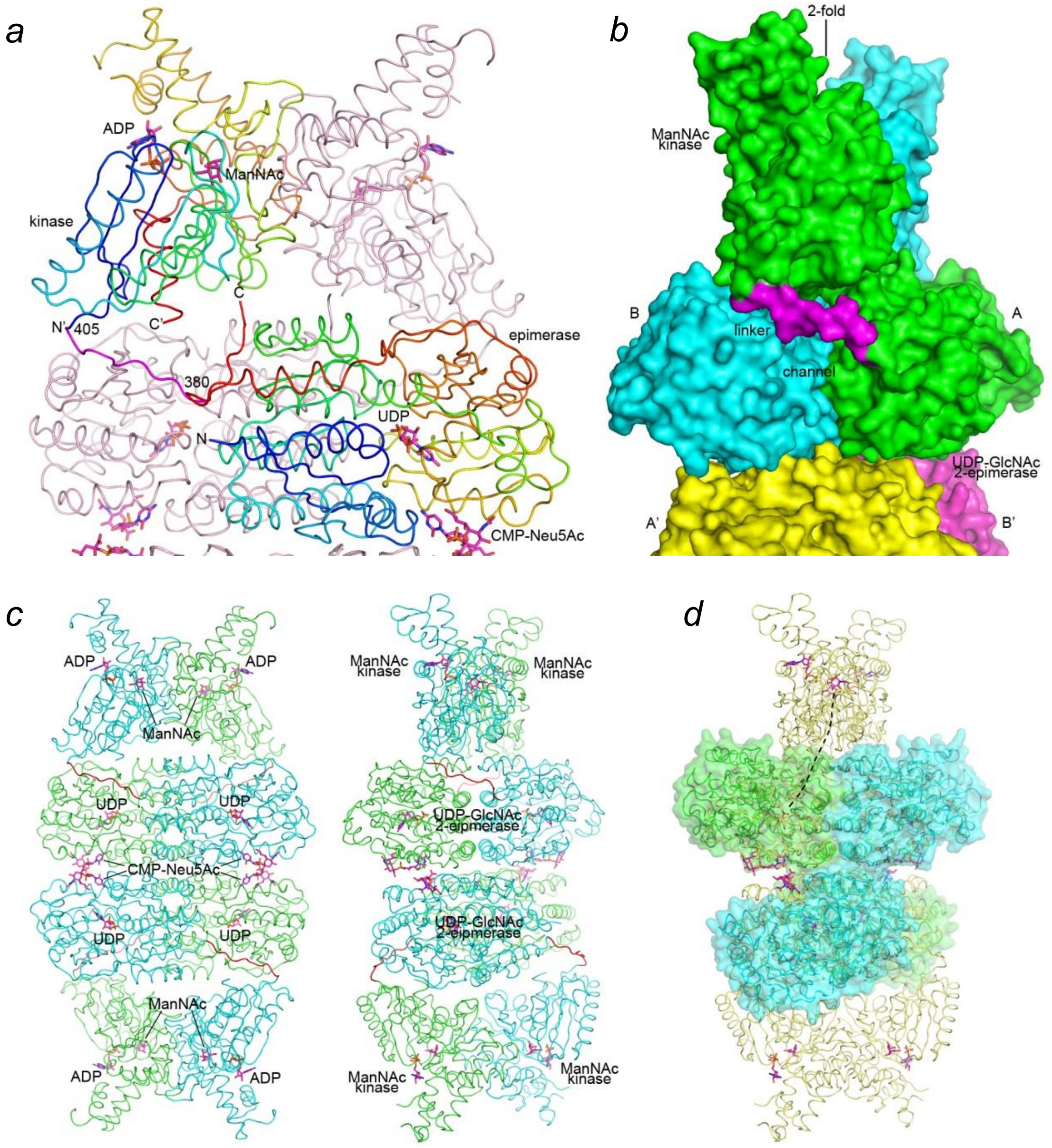

Supplementary Figure S3. The bound ManNAc molecule in monomer C. In (a) the protein is shown as a ribbons diagram and spectrum color coded from N to C terminus. Bound ligands are shown as thick stick models and colored magenta. In (b) the protein model is shown as thin sticks and potential hydrogen bonds to the ManNAc molecule are indicated by pink dashes.

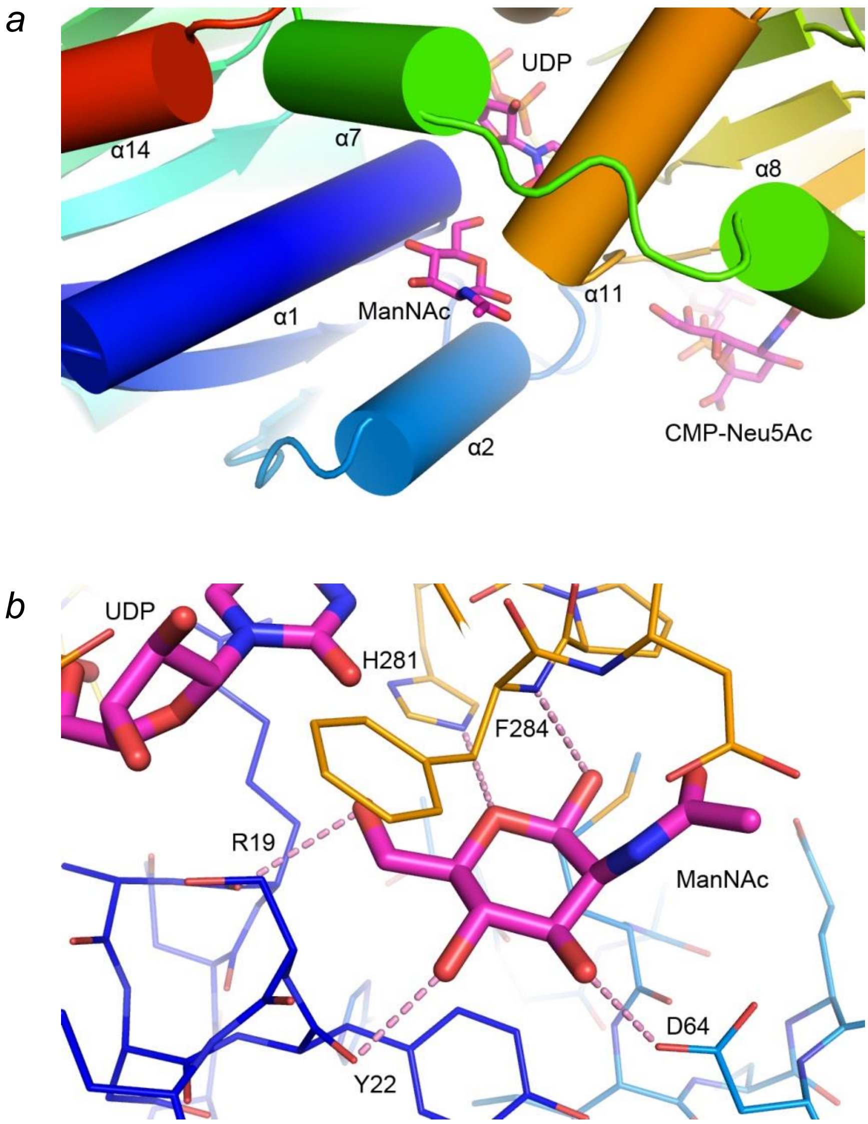

Supplementary Figure S4. Interactions of UDP with non-hydrolyzing epimerase in the active site. The UDP molecule, as well as the allosteric UDP-GlcNAc, is shown as thick sticks model with carbon atoms colored gray. The participating protein residues are shown as thin sticks, and potential hydrogen bonds are indicated by cyan dashes.

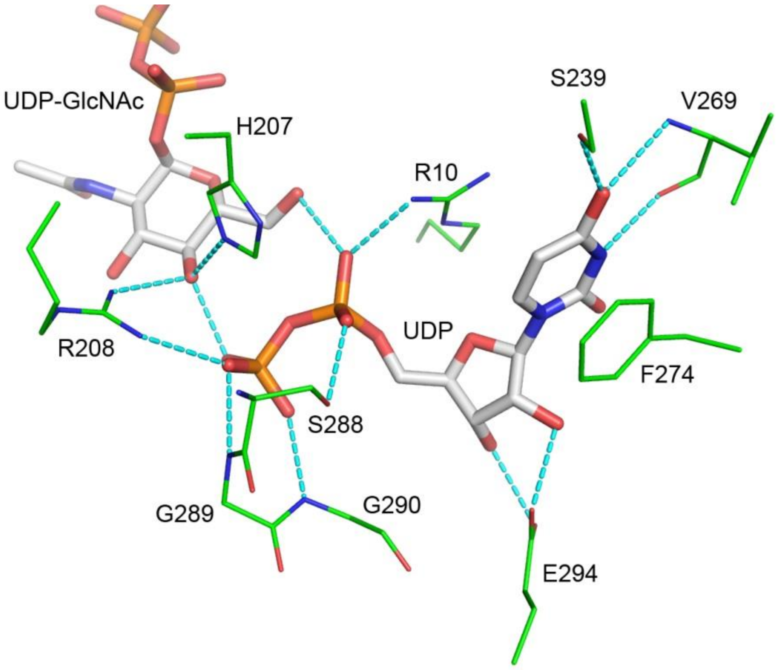

Supplementary Figure S5. Product binding mode. In (a) the crystal structure of non-hydrolyzing *E. coli* enzyme with a bound UDP-ManNAc (PDB 1VGV) is viewed by placing the sugar moiety in the center. The product is shown as a thick stick model with cyan carbons. Some amino acids near ManNAc and the β-phosphate are shown as yellow thin sticks. Hydrogen bonds are depicted with green dashed lines. In (b) the ManNAc moiety from the *E. coli* structure was superimposed on GNE with some adjustments. The original UDP is shown as thick sticks in magenta. Protein residues are shown as thin sticks in green. The hydrogen bonds to the β-phosphate of UDP are indicated by pink dashed lines, whereas some possible bonds to ManNAc are colored light blue.

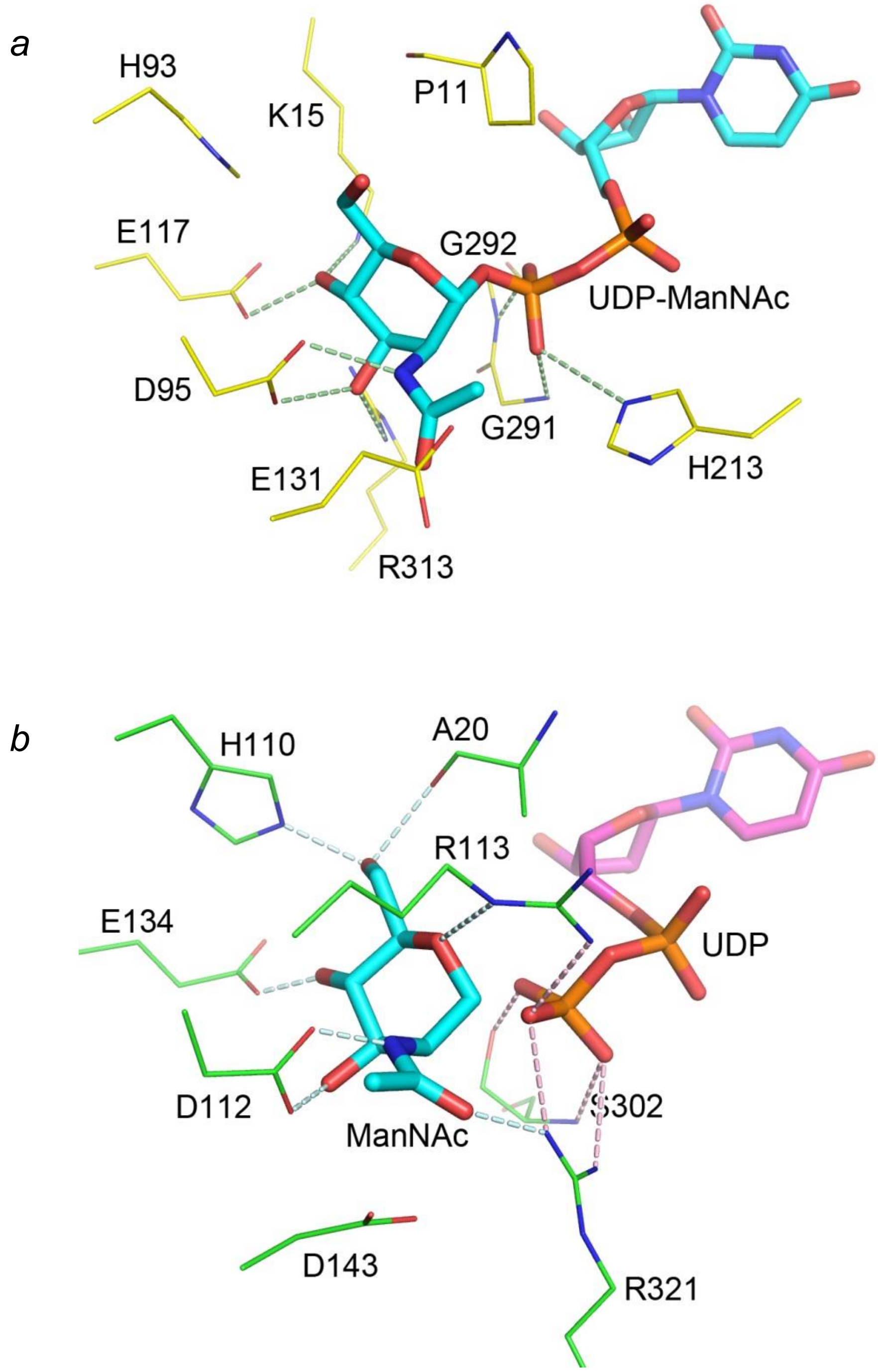

Supplementary Figure S6. Substrate binding mode and catalysis. With some modifications of the ManNAc binding mode, a GlcNAc model is constructed and shown as yellow sticks in (a). UDP is colored magenta. The surrounding amino acid residues are shown as thin green sticks. Potential hydrogen bonds to GlcNAc and UDP are indicated by dashes and colored light yellow and light blue. In (b) the catalytic bases Asp112 and Asp143, as well as the β-phosphate binding Ser302, are colored green. UDP and ManNAc are colored cyan and magenta. Two solvent molecules which may participate in the catalysis are shown as red spheres.

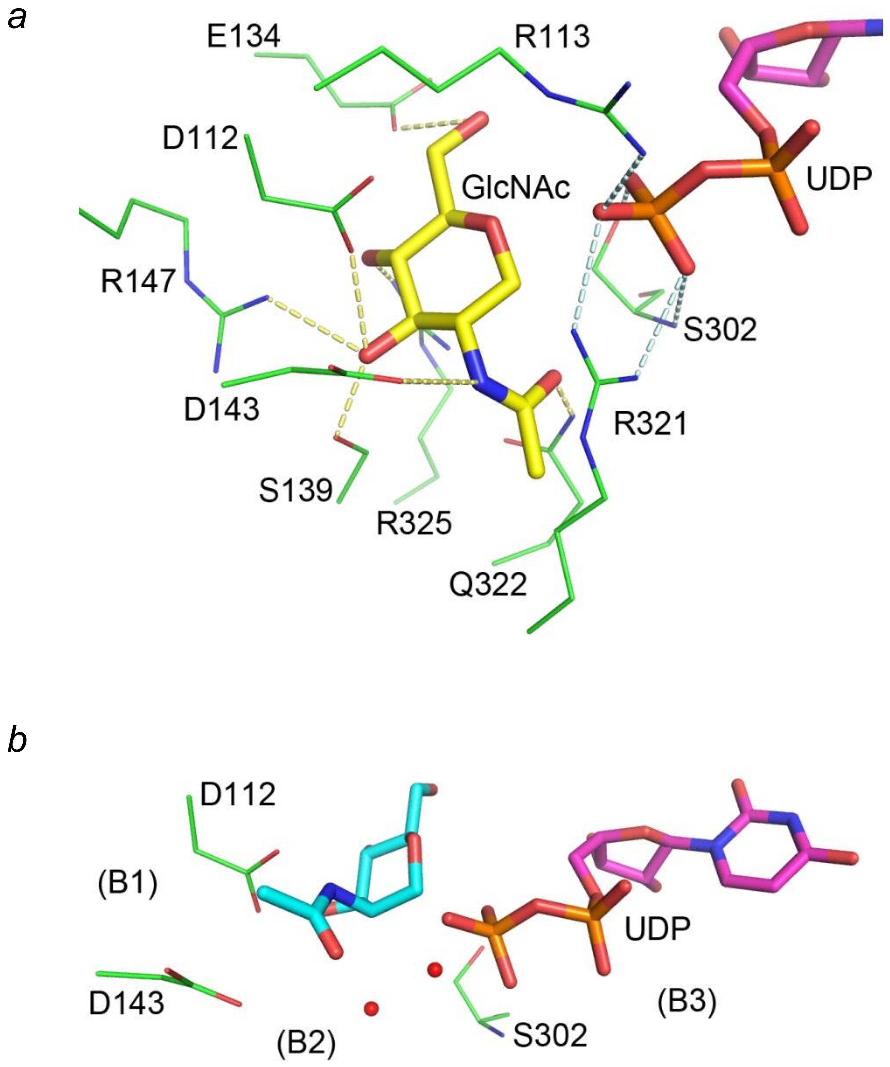

Supplementary Figure S7. Sequence alignment. Residues of human GNE that interact with UDP are marked by blue triangles, whereas those interacting with CMP-Neu5Ac are indicated by red ovals. The enzymes from human, *Neisseria meningitidis* and *Helicobacter pylori* are hydrolyzing epimerases, whereas those from *Bacillus anthracis* and *Methanocaldococcus jannaschii* are non-hydrolyzing. Strictly conserved residues in both classes of epimerase are highlighted by solid red boxes.

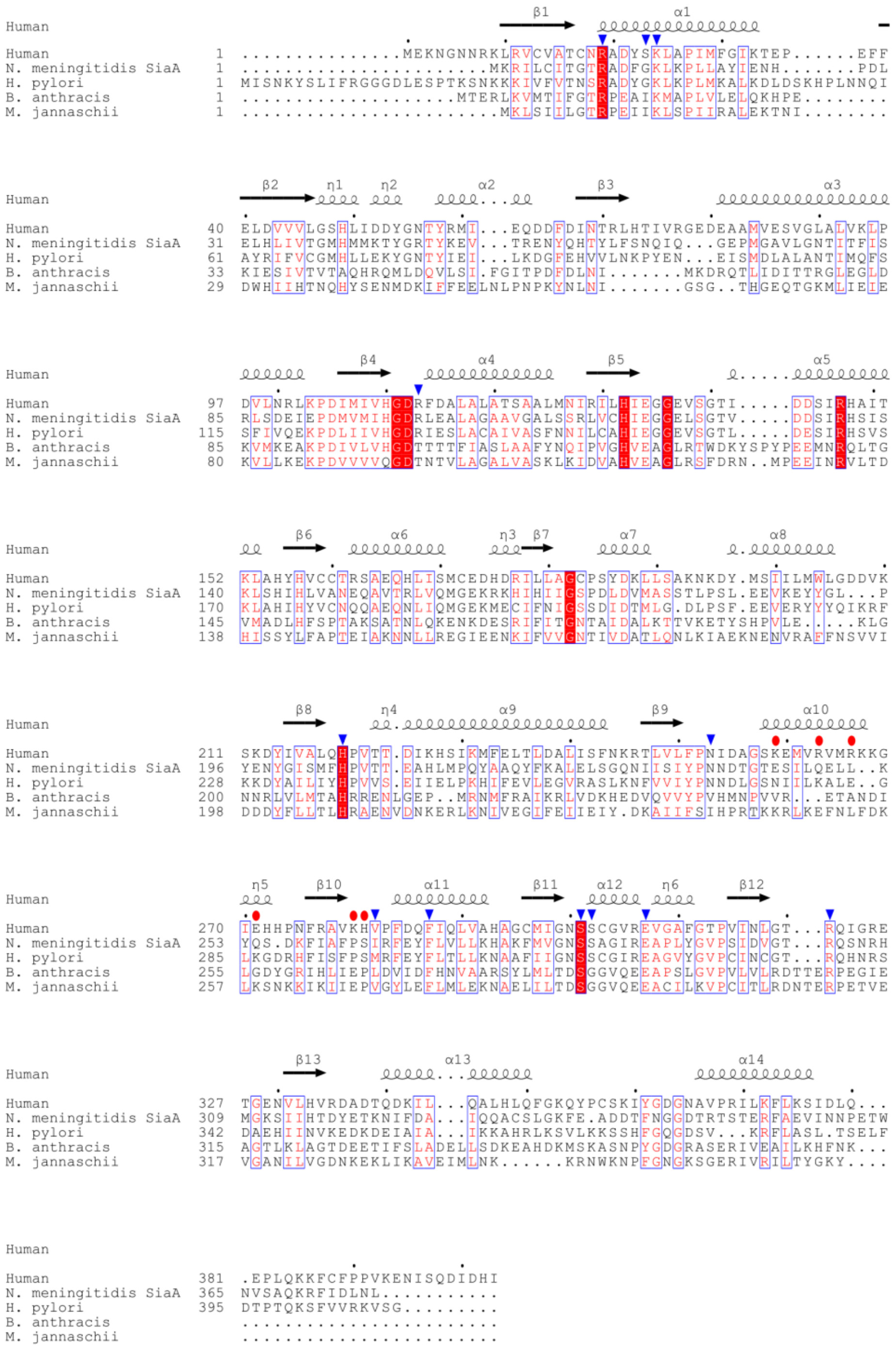

Supplementary Figure S8. Location of HIBM mutants in the full-length GNE model. One monomer is shown as a green ribbon diagram except the mutation sites, which are colored magenta. The other monomers in the same dimer (cyan) and the other dimer (yellow) are shown as surface representations.

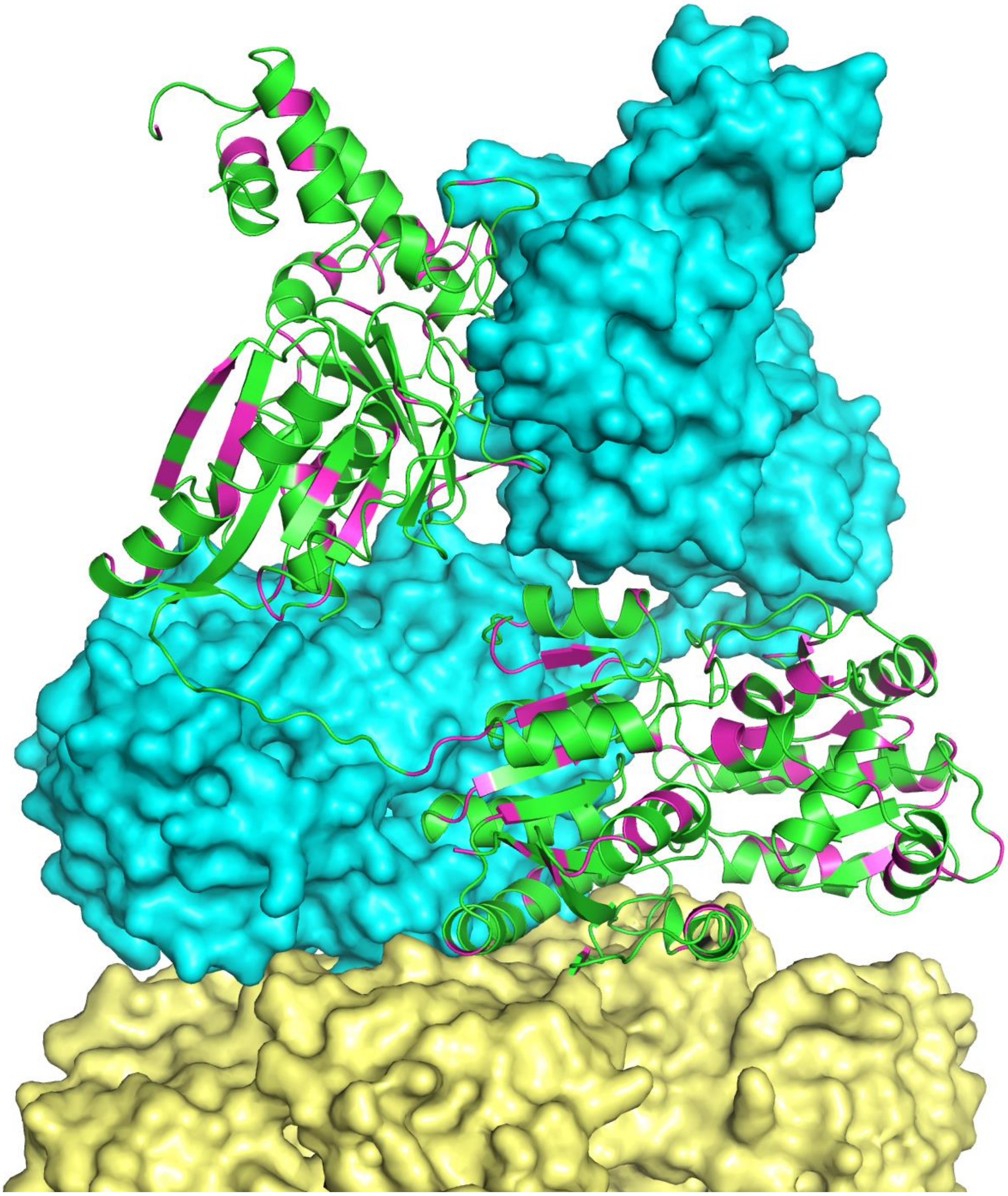

Supplementary Figure S9. Gel-filtration profile and PAGE analysis of the eluted fractions. After purification by using Ni-NTA column, the His-tagged protein was concentrated and loaded onto a Superdex-200 size-exclusion column. The subsequent elution profile is shown in the upper part of this figure, with X-axis and Y-axis indicating the elution volume and the absorbance at 280 nm. The red numbers denote the collected fractions. In the lower part, the contents of selected fractions (indicated on top of the lanes) were analyzed by using polyacrylamide gel electrophoresis (PAGE). On the left side the molecular weights of the marker proteins are indicated (lane M).

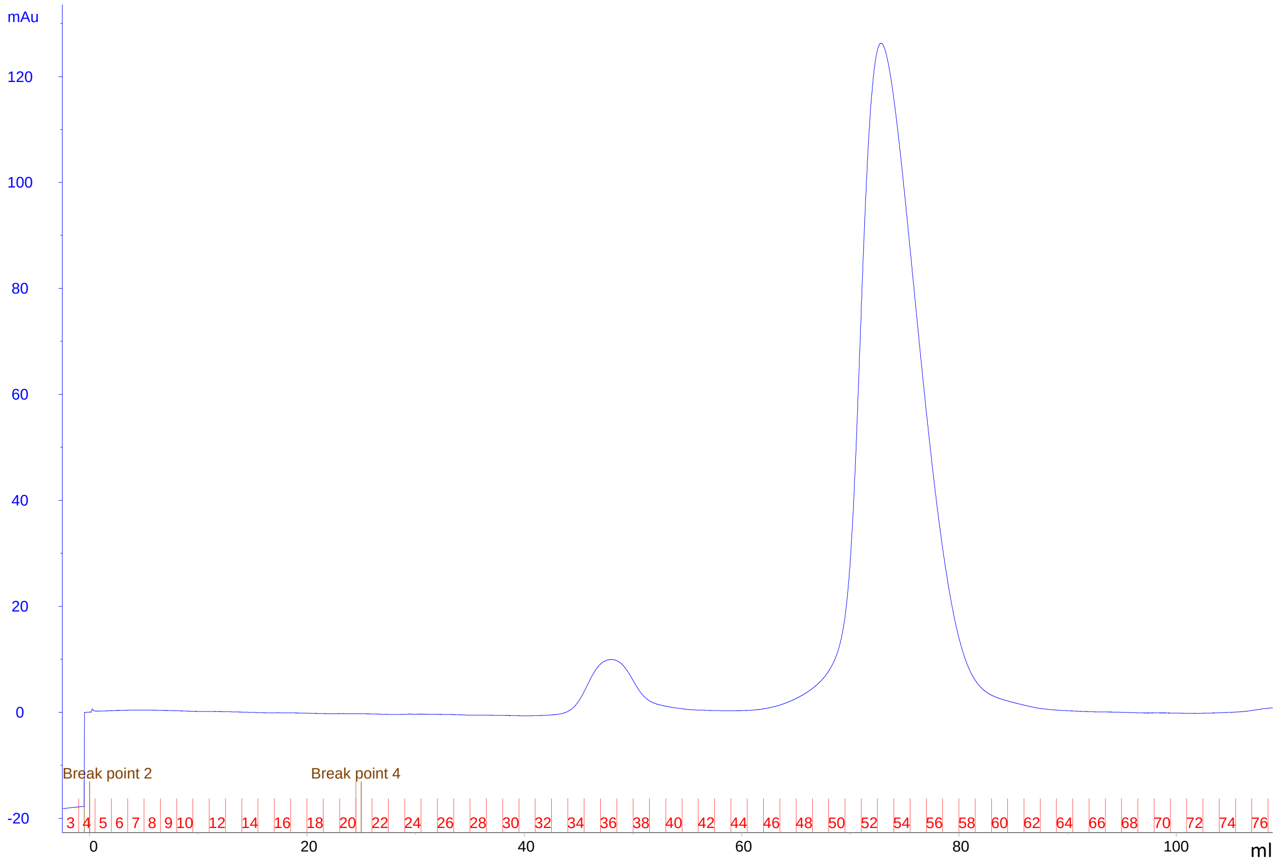

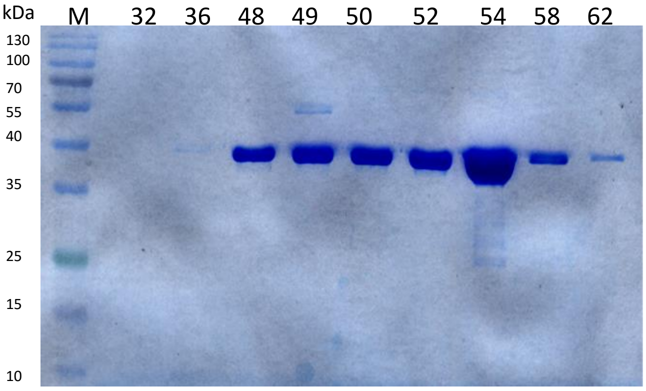

Supplementary Figure S10. Ramachandran plot. There are 17 outliers, most located at the rims of allowed regions. This figure was prepared based on the output of MolProbity.

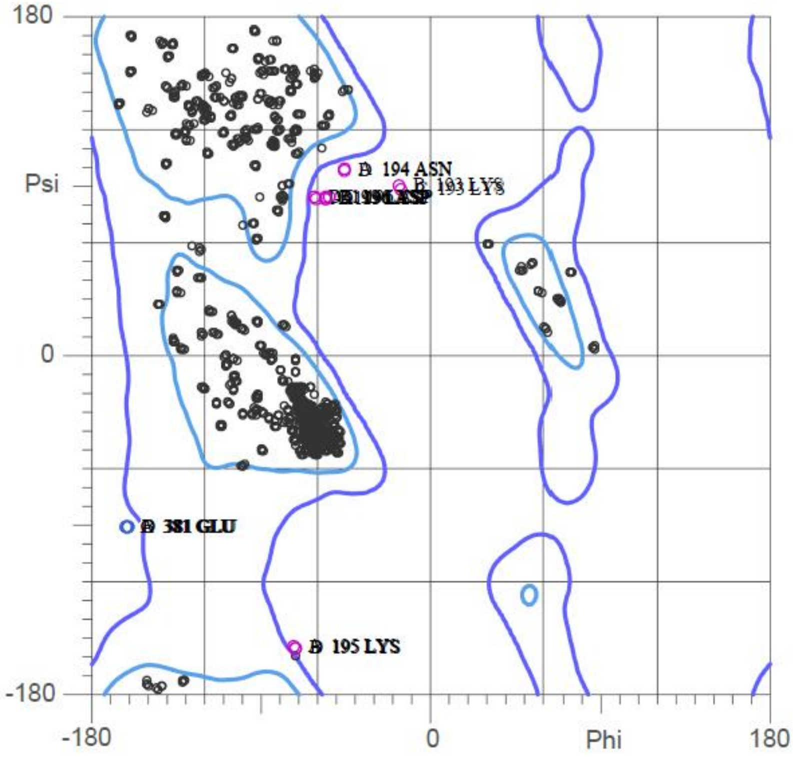

Supplementary Figure S11. The simulated annealing Fo-Fc omit maps are contoured at 4.0 σ and shown as grey meshes around CMP-Neu5Ac (a) and UDP (b).

a

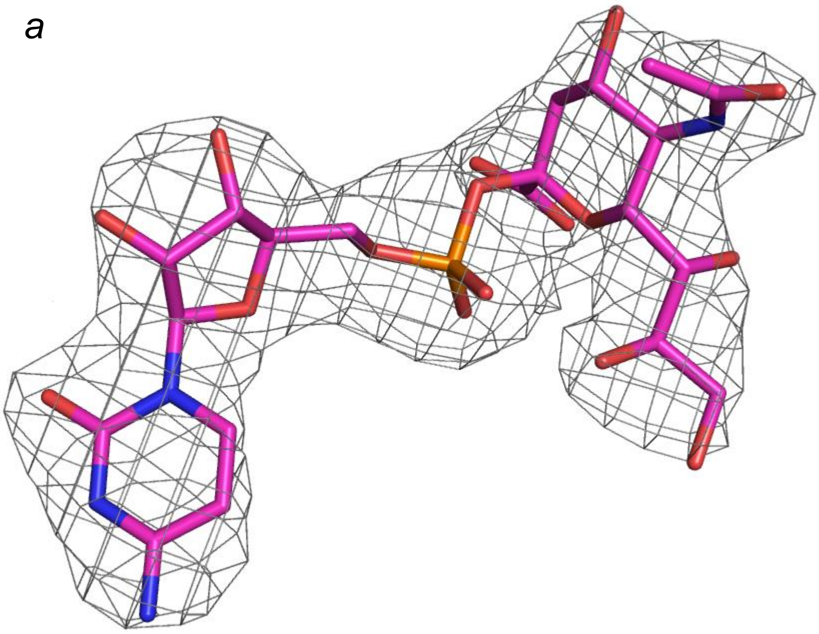

b

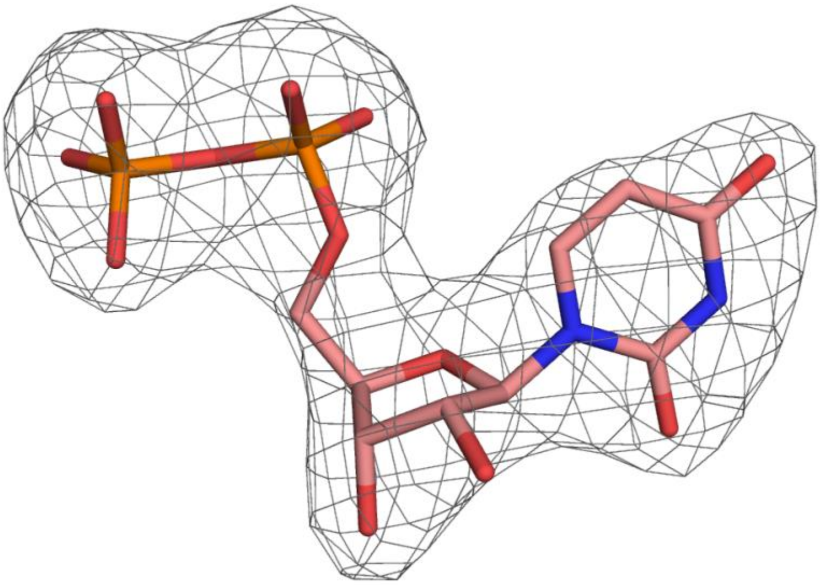

Supporting Reference

1 Saechao, C. *et al.* Novel GNE mutations in hereditary inclusion body myopathy patients of non-Middle Eastern descent. *Genet Test Mol Biomarkers* **14**, 157-162, doi:10.1089/gtmb.2009.0157 (2010).

2 Lu, X., Pu, C., Huang, X., Liu, J. & Mao, Y. Distal myopathy with rimmed vacuoles: clinical and muscle morphological characteristics and spectrum of GNE gene mutations in 53 Chinese patients. *Neurol. Res.* **33**, 1025-1031, doi:10.1179/1743132811Y.0000000070 (2011).

3 Mori-Yoshimura, M. *et al.* Heterozygous UDP-GlcNAc 2-epimerase and N-acetylmannosamine kinase domain mutations in the GNE gene result in a less severe GNE myopathy phenotype compared to homozygous N-acetylmannosamine kinase domain mutations. *J. Neurol. Sci.* **318**, 100-105, doi:10.1016/j.jns.2012.03.016 (2012).

4 Huizing, M. *et al.* Hypoglycosylation of alpha-dystroglycan in patients with hereditary IBM due to GNE mutations. *Mol. Genet. Metab.* **81**, 196-202, doi:10.1016/j.ymgme.2003.11.012 (2004).

5 Kim, B. J. *et al.* Mutation analysis of the GNE gene in Korean patients with distal myopathy with rimmed vacuoles. *J. Hum. Genet.* **51**, 137-140, doi:10.1007/s10038-005-0338-5 (2006).

6 Park, Y. E. *et al.* Limb-girdle phenotype is frequent in patients with myopathy associated with GNE mutations. *J. Neurol. Sci.* **321**, 77-81, doi:10.1016/j.jns.2012.07.061 (2012).

7 Tomimitsu, H. *et al.* Distal myopathy with rimmed vacuoles (DMRV): new GNE mutations and splice variant. *Neurology* **62**, 1607-1610 (2004).

8 Weihl, C. C. *et al.* Novel GNE mutations in two phenotypically distinct HIBM2 patients. *Neuromuscul Disord* **21**, 102-105, doi:10.1016/j.nmd.2010.11.002 (2011).

9 Nalini, A., Gayathri, N., Nishino, I. & Hayashi, Y. K. GNE myopathy in India. *Neurol India* **61**, 371-374, doi:10.4103/0028-3886.117609 (2013).

10 Broccolini, A. *et al.* Novel GNE mutations in Italian families with autosomal recessive hereditary inclusion-body myopathy. *Hum. Mutat.* **23**, 632, doi:10.1002/humu.9252 (2004).

11 Cho, A. *et al.* Mutation profile of the GNE gene in Japanese patients with distal myopathy with rimmed vacuoles (GNE myopathy). *J. Neurol. Neurosurg. Psychiatry* **85**, 914-917, doi:10.1136/jnnp-2013-305587 (2014).

12 Li, H. *et al.* Clinical and molecular genetic analysis in Chinese patients with distal myopathy with rimmed vacuoles. *J. Hum. Genet.* **56**, 335-338, doi:10.1038/jhg.2011.15 (2011).

13 Eisenberg, I. *et al.* Mutations spectrum of GNE in hereditary inclusion body myopathy sparing the quadriceps. *Hum. Mutat.* **21**, 99, doi:10.1002/humu.9100 (2003).

14 Celeste, F. V. *et al.* Mutation update for GNE gene variants associated with GNE myopathy. *Hum. Mutat.* **35**, 915-926, doi:10.1002/humu.22583 (2014).

15 Liewluck, T. *et al.* Mutation analysis of the GNE gene in distal myopathy with rimmed vacuoles (DMRV) patients in Thailand. *Muscle Nerve* **34**, 775-778, doi:10.1002/mus.20583 (2006).

16 Nishino, I. *et al.* Distal myopathy with rimmed vacuoles is allelic to hereditary inclusion body myopathy. *Neurology* **59**, 1689-1693 (2002).
17 Tomimitsu, H. *et al.* Distal myopathy with rimmed vacuoles: novel mutations in the GNE gene. *Neurology* **59**, 451-454 (2002).
18 Sparks, S. E. *et al.* Use of a cell-free system to determine UDP-N-acetylglucosamine 2-epimerase and N-acetylmannosamine kinase activities in human hereditary inclusion body myopathy. *Glycobiology* **15**, 1102-1110, doi:10.1093/glycob/cwi100 (2005).
19 No, D. *et al.* Novel GNE mutations in autosomal recessive hereditary inclusion body myopathy patients. *Genet Test Mol Biomarkers* **17**, 376-382, doi:10.1089/gtmb.2012.0408 (2013).
20 Sim, J. E. *et al.* Clinical characteristics and molecular genetic analysis of Korean patients with GNE myopathy. *Yonsei Med J* **54**, 578-582, doi:10.3349/ymj.2013.54.3.578 (2013).
21 Del Bo, R. *et al.* Novel missense mutation and large deletion of GNE gene in autosomal-recessive inclusion-body myopathy. *Muscle Nerve* **28**, 113-117, doi:10.1002/mus.10391 (2003).
22 Broccolini, A. *et al.* An Italian family with autosomal recessive inclusion-body myopathy and mutations in the GNE gene. *Neurology* **59**, 1808-1809 (2002).
23 Motozaki, Y. *et al.* Hereditary inclusion body myopathy with a novel mutation in the GNE gene associated with proximal leg weakness and necrotizing myopathy. *Eur J Neurol* **14**, e14-15, doi:10.1111/j.1468-1331.2007.01905.x (2007).
24 Grandis, M. *et al.* The spectrum of GNE mutations: allelic heterogeneity for a common phenotype. *Neurol Sci* **31**, 377-380, doi:10.1007/s10072-010-0248-y (2010).
25 Vasconcelos, O. M., Raju, R. & Dalakas, M. C. GNE mutations in an American family with quadriceps-sparing IBM and lack of mutations in s-IBM. *Neurology* **59**, 1776-1779 (2002).
26 Eisenberg, I. *et al.* The UDP-N-acetylglucosamine 2-epimerase/N-acetylmannosamine kinase gene is mutated in recessive hereditary inclusion body myopathy. *Nat. Genet.* **29**, 83-87, doi:10.1038/ng718 (2001).
27 Ro, L. S. *et al.* Phenotypic variability in a Chinese family with rimmed vacuolar distal myopathy. *J. Neurol. Neurosurg. Psychiatry* **76**, 752-755, doi:10.1136/jnnp.2004.048876 (2005).
28 Chu, C. C. *et al.* Heterozygous mutations affecting the epimerase domain of the GNE gene causing distal myopathy with rimmed vacuoles in a Taiwanese family. *Clin. Neurol. Neurosurg.* **109**, 250-256, doi:10.1016/j.clineuro.2006.09.008 (2007).
29 Darvish, D., Vahedifar, P. & Huo, Y. Four novel mutations associated with autosomal recessive inclusion body myopathy (MIM: 600737). *Mol. Genet. Metab.* **77**, 252-256 (2002).
30 Stober, A. *et al.* Novel missense mutation p.A310P in the GNE gene in autosomal-recessive hereditary inclusion-body myopathy/distal myopathy with rimmed vacuoles in an Italian family. *Neuromuscul Disord* **20**, 335-336, doi:10.1016/j.nmd.2010.02.013 (2010).

31 Chai, Y., Bertorini, T. E. & McGrew, F. A. Hereditary inclusion-body myopathy associated with cardiomyopathy: report of two siblings. *Muscle Nerve* **43**, 133-136, doi:10.1002/mus.21839 (2011).

32 Leroy, J. G. *et al.* Dominant inheritance of sialuria, an inborn error of feedback inhibition. *Am. J. Hum. Genet.* **68**, 1419-1427, doi:10.1086/320598 (2001).

33 Behin, A. *et al.* [Distal myopathy due to mutations of GNE gene: clinical spectrum and diagnosis]. *Rev Neurol (Paris)* **164**, 434-443, doi:10.1016/j.neurol.2008.02.040 (2008).

34 Kannan, M. A. *et al.* Distal myopathy with rimmed vacuoles and inflammation: a genetically proven case. *Neurol India* **60**, 631-634, doi:10.4103/0028-3886.105199 (2012).

35 Tasca, G. *et al.* Muscle imaging findings in GNE myopathy. *J Neurol* **259**, 1358-1365, doi:10.1007/s00415-011-6357-6 (2012).

36 Fisher, J., Towfighi, J., Darvish, D. & Simmons, Z. A case of hereditary inclusion body myopathy: 1 patient, 2 novel mutations. *J Clin Neuromuscul Dis* **7**, 179-184, doi:10.1097/01.cnd.0000211406.94445.f0 (2006).

37 Krause, S. *et al.* A novel homozygous missense mutation in the GNE gene of a patient with quadriceps-sparing hereditary inclusion body myopathy associated with muscle inflammation. *Neuromuscul Disord* **13**, 830-834 (2003).

38 Amouri, R. *et al.* Allelic heterogeneity of GNE gene mutation in two Tunisian families with autosomal recessive inclusion body myopathy. *Neuromuscul Disord* **15**, 361-363, doi:10.1016/j.nmd.2005.01.012 (2005).
